# Trait specialization facilitates autonomous selfing ability in a mixed‐mating plant

**DOI:** 10.1002/ajb2.70095

**Published:** 2025-09-04

**Authors:** Hanna Makowski, Keric Lamb, Austin M. Kim, Emily Scott, Laura F. Galloway

**Affiliations:** ^1^ Department of Biology University of Virginia P.O. Box 400328 Charlottesville 22904 Virginia USA

**Keywords:** autonomous selfing, *Campanula americana*, floral traits, mixed‐mating, pollen‐collecting hairs, secondary pollen presentation, self‐fertilization

## Abstract

**Premise:**

Transitions from outcrossing to selfing often drive the evolution of floral traits in a predictable way. However, these expectations are not as straightforward for mixed‐mating systems. In this study, we examine variation in pollen‐collecting hairs, a floral structure involved in secondary pollen presentation within Campanulaceae. While secondary pollen presentation is hypothesized to have evolved to promote outcrossing, we evaluate the association of pollen‐collecting hairs with selfing ability.

**Methods:**

We characterized pollen‐collecting hair morphology and retraction phenology in 15 populations of *Campanula americana* with known variation in self‐fertilization ability using time‐series collections and automated image analysis of pollen‐collecting hair length.

**Results:**

There was two‐fold variation in the length of pollen‐collecting hairs across populations that was associated with a population's within‐flower selfing ability. Retraction rate of pollen‐collecting hairs also varied among populations and was associated with selfing ability. Populations with greater selfing ability had longer hairs that retracted quickly early in floral anthesis.

**Conclusions:**

We show pollen‐collecting hairs, a trait thought to have evolved to promote outcrossing, is associated with within‐flower selfing ability. Through developmental changes in length, pollen‐collecting hairs appear to be a plastic phenotype that is both associated with autonomous selfing and with outcrossing in *C. americana*. This provides support for trait specialization rather than trade‐offs, and for the ‘best of both worlds’ hypothesis of mixed mating‐system evolution.

Animal‐pollinated flowers typically have multiple attractive traits to promote outcrossing through pollinator visitation (Eckhart, 1991; Raguso, 2004), as well as morphological (Webb and Lloyd, 1986; Routley et al., 2004) and genetic mechanisms (Gerstel, 1950; Hiscock and McInnis, 2003; Takayama and Isogai, 2005) to prevent self‐fertilization. The evolution of self‐fertilization is often accompanied by a reduction in these attractive traits (Ornduff, 1969; Goodwillie et al., 2010) as well as the loss or weakening of morphological and genetic self‐incompatibility (Lloyd, 1965; Ritland and Ritland, 1989; Brunet and Eckert, 1998; Igic et al., 2008), a suite of traits known as the selfing syndrome. However, the distribution of plant mating systems, the degree to which reproduction occurs through outcrossing or selfing, is not bimodal and about 33% of plant species both outcross and self in mixed‐mating systems (Vogler and Kalisz, 2001). Mixed‐mating systems confer reproductive assurance via selfing without sacrificing the potential benefits of outcrossing (Kalisz and Vogler, 2003). While the selfing syndrome is well‐defined for transitions to a predominately selfing mating system, less is known about the traits that facilitate selfing in mixed‐mating systems.

Self‐fertilization within a flower without an insect visitor (i.e., autonomous selfing) is a common form of reproductive assurance. For autonomous selfing to occur, the male and female sexual phases must overlap. Morphology and phenology of structures associated with pollen presentation and stigmatic accessibility and receptivity can influence the amount and timing of sexual phase overlap. Selfing is often associated with a reduction in the spatial (Belaoussoff and Shore, 1995; Takebayashi et al., 2006; Toräng et al., 2017; Fishman et al., 2022) and temporal separation of the sexes within a flower (Kalisz et al., 2011; Koski et al., 2018), leading to increased sexual phase overlap (Totland and Schulte–Herbrüggen, 2003; Brys et al., 2013). This overlap typically comes at the detriment of outcrossing ability in predominately selfing species. However, traits that facilitate sexual phase overlap and thus reproductive assurance in mixed‐mating taxa are poorly understood. Mechanisms of sexual phase overlap in mixed‐mating systems may have been overlooked because they occur in plants that appear to be adapted for outcrossing (Kalisz et al., 1999).

Secondary pollen presentation, the relocation of pollen from the anthers to elsewhere in the flower, is hypothesized to have evolved to enhance outcrossing. This relocation of pollen may increase outcrossing by extending the length of male phase, optimizing the placement of pollen on biotic vectors, and prolonging pollen viability (Shetler, 1979; Howell et al., 1993; Nyman, 1993a, 1993b; Yeo, 1993; Westerkamp and Weber, 1997). Through altering the spatial separation of male and female sexual function, secondary pollen presentation can lead to intersexual interference (Castro et al., 2008), however there are often compensatory mechanisms to prevent self‐fertilization, i.e., self‐incompatibility, dichogamy (Howell et al., 1993; Lin et al., 2012). In the Campanulaceae, pollen is relocated from the anthers to hairs along the style (Howell et al., 1993; Yeo, 1993). Flowers are typically protandrous with pollen presented on the hairs at anthesis. Over time, the hairs retract, gradually releasing pollen and making it accessible for transfer (Carolin, 1960). After the hairs have retracted, the stigma matures and flowers transition to female phase. Early descriptive work in *Campanula* posits that gradual hair retraction spreads pollen dispersal across multiple visitors, and touch‐sensitive retraction times stigmatic maturation to follow pollen dispersal, both promoting outcrossing (Nyman, 1993b). However, there has been much debate about the adaptive significance of the hairs (Shetler, 1979; Nyman, 1993a, b).

While most species in the Campanulaceae are self‐incompatible and obligately outcrossing, mixed mating has repeatedly evolved in the family (Roquet, 2008). Due to pollen‐collecting hairs' direct influence on pollen dispersal schedules they may influence pollen retention into the female phase and therefore selfing potential in mixed‐mating taxa. Through this mechanism, pollen‐collecting hairs, thought to have evolved to promote outcrossing (Nyman, 1993a, 1993b), may also facilitate selfing. Therefore, pollen‐collecting hairs provide the opportunity to test whether single traits may both facilitate selfing and outcrossing, and thus play a key role in mixed‐mating systems.

*Campanula americana* L. (=*Campanulastrum americanum* Small), a self‐compatible but largely outcrossing herb (Galloway et al., 2003; Koski et al., 2019b), is an excellent system for exploring the role of secondary pollen presentation in mixed‐mating systems. The species has protandrous flowers that transition to female phase 2–4 days after anthesis in the absence of pollinators, allowing for autonomous selfing (Koski et al., 2018). The potential for autonomous selfing (fruit set in pollinator free environment) varies geographically and is greater in northern and western populations of its Eastern North American range (Koski et al., 2017). There is an increased speed of transition to female phase, retention of pollen into the female phase, pollen deposition on the stigma in the absence of pollinators, and production of autonomous fruit earlier in floral phenology in populations with high autonomous selfing potential compared to those with lower selfing potential (Koski et al., 2018; Leibman et al., 2018). The process by which self‐fertilization occurs is not known. Because pollen‐collecting hairs hold onto and then release pollen for transfer, we hypothesize that they could play a role in autonomous selfing. High autonomous selfing ability may be associated with longer hairs and/or hairs that retract more slowly, thereby allowing them to retain more pollen into stigmatic receptivity, increasing the potential for autonomous self‐fertilization.

The goal of this study was to determine the association between the mechanism of secondary pollen presentation—specifically pollen‐collecting hairs—and mating system in *C. americana*. To do this we determined the variation among populations in the length of pollen‐collecting hairs, the phenology of hair retraction over floral anthesis, and their association with autonomous selfing. We use multiple populations with known autonomous selfing potential to ask: (Q1) Do pollen‐collecting hairs vary in length or number among populations at flower opening and (Q2) is variation associated with autonomous selfing ability? (Q3) What is the timing of retraction of the hairs and (Q4) and is retraction associated with autonomous selfing?

## MATERIALS AND METHODS

### Study system

**Figure 1:**
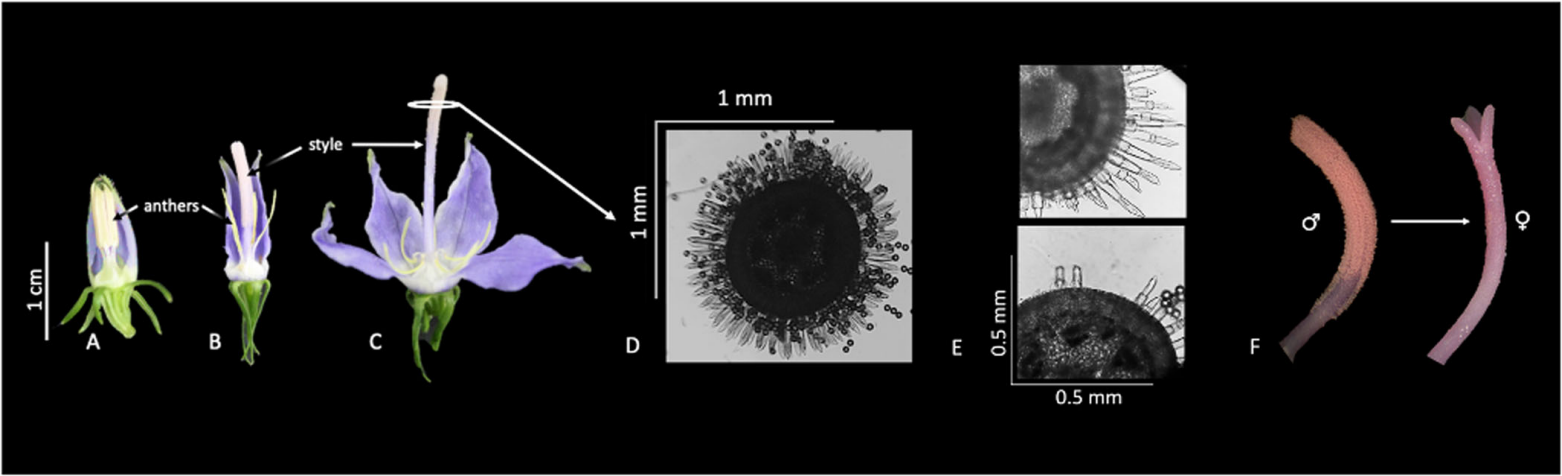
Secondary pollen presentation in *Campanula americana*. First, (A) pollen is deposited from the anthers onto hairs along the style while the flower is in bud. The flower opens and the style begins to elongate (B and C). Pollen is present on hairs along a cross section of the style (D). Over time, the hairs retract (E), the stigmatic lobes open and the flower becomes functionally female (F).

As in other Campanulaceae, pollen is presented on pollen‐collecting hairs in the herb *Campanula americana* L. (=*Campanulastrum americanum* Small). Anthers dehisce in the bud and pollen is deposited onto adjacent pollen‐collecting hairs along the outer surface of the style (Figure 1A, D, E). Upon anthesis, the style elongates, and the flower displays the pollen sub‐terminally along the style (Figure 1B, C; Erbar and Leins, 1990). Pollen is held tightly by hairs; over time the pollen‐collecting hairs gradually retract into the style in a telescoping fashion, releasing pollen for transfer (Figure 1E; Carolin, 1960; Nyman, 1993a). The stigmatic lobes open and become receptive after most of the hairs have retracted (Figure 1F).

*Campanula americana* is insect‐pollinated and largely outcrossing though fully self‐compatible (*t*_*m*_ = 0.81; Galloway et al., 2003; Koski et al., 2019b). The flowers open in male phase around noon (Evanhoe and Galloway, 2002). When female phase begins, the tip of the style splits into three lobes, the stigma becomes accessible, and over time the stigmatic lobes curl down towards the style. If pollinators are not present, the transition to female phase and therefore the opportunity to self‐fertilize occurs 2 to 4 days after anthesis (Evanhoe and Galloway, 2002; Koski et al., 2018). The hairs are touch sensitive and when manipulated, typically by pollinators, the transition to female phase is expedited (*Campanula*, Nyman, 1993a; *C. americana*, Koski et al., 2018). The extent to which manipulation of pollen‐collecting hairs accelerates the transition to female phase is positively correlated with a population's autonomous selfing potential (Koski et al., 2018).

There is a clinal increase in autonomous selfing potential across *C. americana*'s eastern North America range (Koski et al., 2017), with populations in the northern and western reaches having the greatest selfing potential. Although reduced herkogamy by the curling of the stigmatic lobes to increase proximity to pollen on the outer surface of the style is hypothesized to be a method of autonomous selfing in Campanulaceae (Shetler, 1979; Stephenson et al., 2000; Vranken et al., 2014), it is not associated with autonomous selfing ability in *C. americana* (Koski et al., 2018). Selfing potential is associated with floral phenology in *C. americana*, with a shorter male phase in populations with greater autonomous selfing potential, i.e., reduced dichogamy (Koski et al., 2018).

### Sampling scheme and collection

To assess morphological variation in pollen‐collecting hairs in *C. americana*, we sampled 15 populations from a latitudinal cline across the species’ range (Appendix S1). These populations varied in their tendency to autonomously self, measured as the proportion of flowers that produce seed‐bearing fruit in a pollinator‐free greenhouse. Autonomous selfing in sampled populations was measured in previous studies and ranged from 0.28 to 0.68 (Koski et al., 2017, 2018; Makowski et al., 2024a; H. Makowski, unpublished data; Appendix S1). Seeds from multiple maternal families from each population were sown in a 3:1 mixture of PGX soilless growth media (BFG Supply, Burton, Ohio, USA) and calcinated clay particles. Germination occurred in a growth chamber at 21°C/14°C (day/night) with 12‐hour days. Six weeks after sowing, seedlings were transferred to a cold room (5°C, 12‐hour days) for seven weeks of vernalization to cue flowering. Individuals were then transplanted into cone‐tainers (Stuewe & Sons, Inc., Tangent, Oregon, USA) and placed in the University of Virginia greenhouse where lights extended daylength to 16 hours. We grew 54 plants from 10 populations in 2021 (mean 4.6 maternal families/population, range 1–10), and 154 plants from 11 populations in 2022 (mean 7.5 maternal families/population, range 1–14). Six populations were sampled in both years.

We sampled styles at flower opening to assess initial pollen‐collecting hair length and at a series of times after opening to evaluate retraction pattern. Sampled flowers were tagged in bud in the morning before 9am and then collected at flower opening (timepoint 0) and 6‐, 9‐, or 21 hours after flowers opened. Collected styles were fixed in FAA solution (2:10:1:5 v:v:v:v, 37% formaldehyde: 95% ethanol: 100% acetic acid: H_2_O). The 2021 style samples include only the initial timepoint where pollen‐collecting hairs were fully extended (timepoint 0; 73 samples from 54 individuals; repeat samples from an individual were averaged). The 2022 plants were sampled at timepoint 0 as well as 6, 9, and/or 21‐hour timepoints from the same individuals. Nine populations were sampled at all timepoints, and two populations only at timepoint 0. Only populations with all timepoints sampled were included for retraction analysis (total 484 samples; 13.4 samples/population/timepoint on average). However, the same individuals were not necessarily sampled at each of the later timepoints.

### Sample dissection and imaging

**Figure 2:**
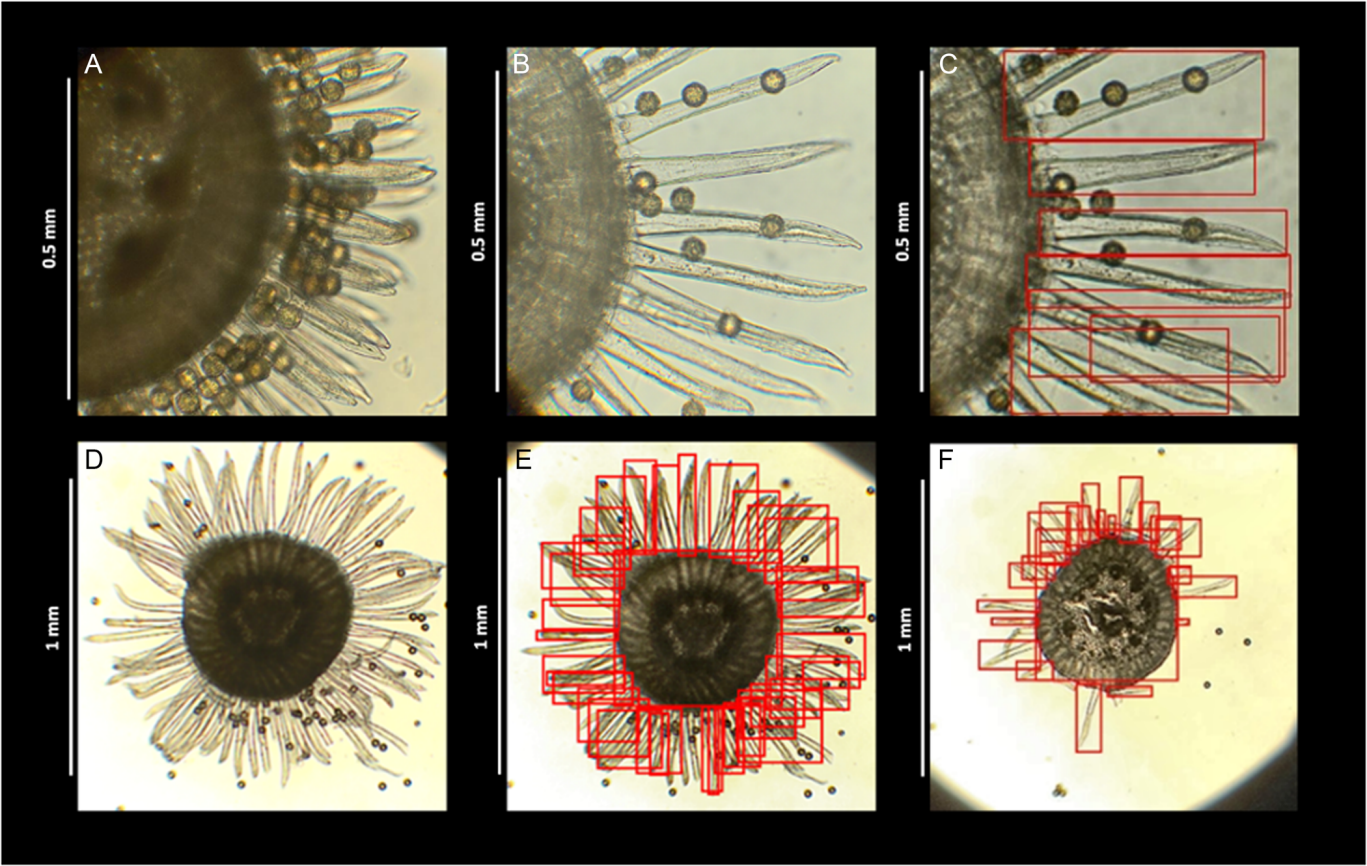
A cross‐sectional view of *Campanula americana*'s style and pollen‐collecting hairs. A 200× view of a low‐autonomy population, AL79 (A), and a high‐autonomy population, KS60 (B), with an example of the AI image detection model output for the 200× images used to get average hair length (C). Hairs do not retract evenly around the style, so we imaged at 40× to get the whole style in view. A high‐autonomy population, IA18 is shown at timepoint 0 (D) and with AI model output (E), and 6 hours after floral anthesis (F).

Style samples were dissected and imaged to view the pollen‐collecting hairs. Styles were first vortexed and rinsed with additional fixative to remove pollen. Three to four thin (<0.5 mm) cross‐sectional slices were made just below the split of the three stigmatic lobes with a 15° stab knife on a dissecting microscope. The thinnest slice with the least amount of pollen in view was imaged on a standard light microscope. For the initial timepoint samples in 2021, we imaged pollen‐collecting hairs at 200× using a camera attached to a light microscope and captured approximately 25% of the circumference of each style in the image (Figure 2A, B). In 2022, we also included 40× images with the entire style circumference in addition to the 200× images (Figure 2D). A ruler was imaged at both magnifications to convert pixels to metric units.

### Image processing using computer vision and object detection

To obtain data on pollen‐collecting hair count and length, we trained two models to detect hairs, one for 40× images and the other for 200× images. We performed model training and object detection using Detecto (Detecto n.d.), that implements PyTorch (Paszke et al., 2019) for custom model development using Faster Region‐based Convolutional Neural Networks (Ren et al., 2016). To develop the two models, we used 107 images at 40× and 506 images at 200×. For each image we drew bounding boxes around each pollen‐collecting hair using Make Sense AI (Skalski 2019) to create an annotated image set (Figure 2). We then applied contrast‐limited adaptive histogram equalization to make hairs more visually prominent, and divided the annotated images for each model, i.e., 40× and 200×, into two sets. One set (70%) was used to train the models and the other set (30%) was for validation during model training. We ran the models for 25 epochs, with a learning step size of 5 and a learning rate of 1 × 10^−3^.

Following model development, we used a small subset of the experimental dataset (around 30 images) to determine the model accuracy. We regressed hand‐counts of pollen‐collecting hair number in each image against model outputs, yielding R^2^ = 0.87 for the 40× model and R^2^ = 0.80 for the 200× model, indicating strong performance of the models. Visual inspection of hand‐counts and model hair identification determined that the model performed well and made better calls in some cases. Finally, the correlation between hand‐counts done by different people were more consistent in the 40× image assessment (R^2^ = 0.96) and less consistent in the 200× assessment (R^2^ = 0.74) than those between people and the image detection models. All experimental images (below) were visually confirmed with model output to check for erroneous data (e.g., detached hairs, extra style tissue with hairs).

We ran the models on the experimental image dataset of 484 40× and 144 200× images to obtain data for analyses. For both the 40× and 200× models, the output was the number and dimensions of boxes drawn around individual pollen‐collecting hairs. Because the 40× and 200× images showed different proportions of the style, we used different approaches to get data for analysis. Pollen‐collecting hair number was estimated as the number of bounding boxes from the 40× images, where the full style cross‐section was in view. The length of each pollen‐collecting hair was estimated as the hypotenuse of each hair's bounding box for both 40× and 200× images (Figure 2). Lengths were converted from pixels to metric units using Image‐J (Schneider et al., 2012, 40× = 15.26 pixels/0.05 mm; 200× = 13.14 pixels/0.01 mm) and then average hair length was calculated for each sample. The 40× images, with the full circumference of the style, were used to assess average length of pollen‐collecting hairs over retraction. After timepoint 0, some hairs had fully retracted, i.e., zero length, which we accounted for using a weighted average hair length. Weighted average hair length was the sum of the length of the hairs in an image divided by the average number of hairs at the initial timepoint for that population.

### Analysis

We determined if populations were differentiated for pollen‐collecting hair traits, the phenology of retraction, and the association of hair traits with autonomous selfing ability. Residuals of all variables met assumptions of normality and homoscedasticity. To determine if the length of pollen‐collecting hairs varied across populations (Q1), we used the 200× initial pollen‐collecting hair length data (timepoint 0) from both years of data collection. This included 190 samples from 144 individuals across 15 populations with six of these populations replicated between years. We modeled an individual's average initial pollen‐collecting hair length as a function of population, with year of data collection included as a random effect (R, “lmer”). To determine if the initial number of pollen‐collecting hairs varied across populations, we used the timepoint 0 values from the 2022 40× dataset (84 individuals, 11 populations). We also used this dataset to test whether initial hair number and length (timepoint 0) were correlated.

To evaluate whether pollen‐collecting hair length or number at flower opening was associated with autonomous selfing ability (Q2), we modeled the population average pollen‐collecting hair length (200× dataset) and number (40× dataset) as a function of the population's autonomous selfing ability. Population averages were weighted by number of individuals included in the average.

We assessed retraction phenology (Q3) using the number and length of pollen‐collecting hairs in the nine populations where individuals were repeatedly sampled across timepoints (40× images, 2022 dataset). Because the estimated pollen‐collecting hair length at later timepoints used the average number of hairs per population at timepoint 0 in the denominator, we calculated averages for each population at each timepoint and used population as our level of replication for analyses. We analyzed retraction across timepoints by modeling the effect of timepoint in ANOVA using population averages for pollen‐collecting hair number and estimated average hair length as response variables, with population as a random effect. We used post‐hoc Tukey tests to compare timepoints.

To assess differences in retraction across autonomous selfing ability (Q4), we used an ANOVA with the estimated average pollen‐collecting hair length from the 40× timing data set (same as Q3) as a response variable (R, “lmer”), with timepoint, autonomous selfing, and their interaction as fixed effects. Population was included as a random effect. We only included the first two timepoints in this analysis after finding that most of the retraction happens between 0 and 6 hours (Q3).

## RESULTS

**Figure 3:**
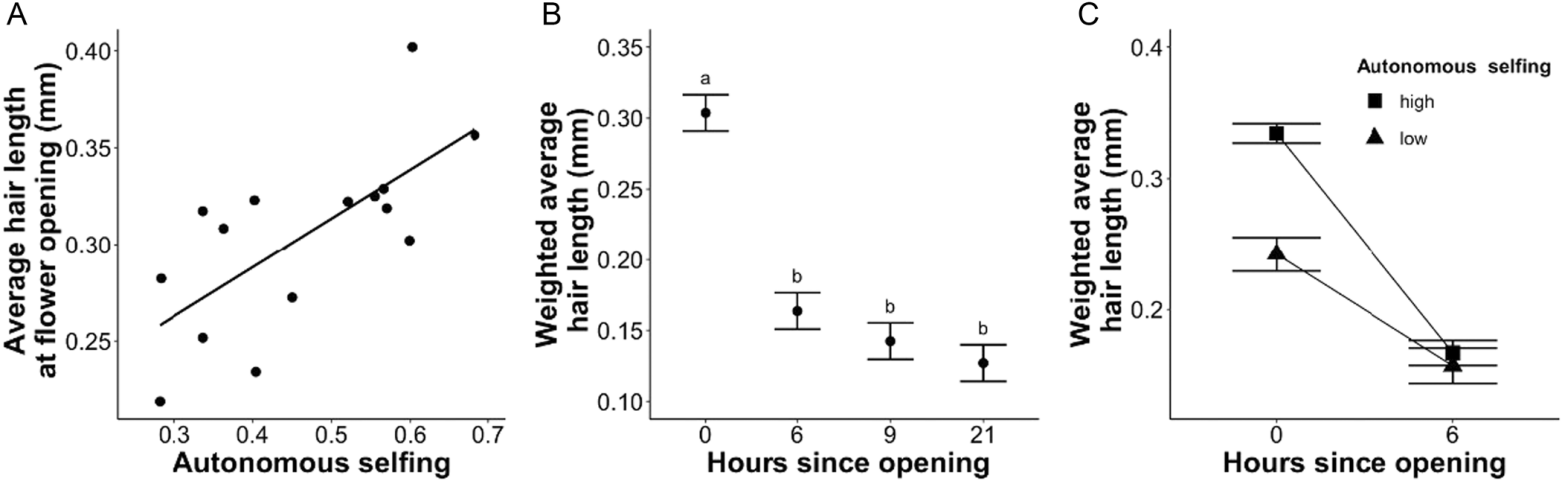
Pollen‐collecting hair length and retraction patterns in *Campanula americana*. (A) Average pollen‐collecting hair length for 15 populations of *C. americana* across a range of autonomous selfing values. (B) The weighted average length of *C. americana* pollen‐collecting hairs over the first day of floral anthesis. Points represent averages across nine populations at each timepoint. Error bars are SE. Letters signify statistical difference from post‐hoc Tukey test. (C) Weighted average pollen‐collecting hair length at floral opening and 6 hours after floral anthesis. Statistics for (C) were run on continuous values for autonomous selfing but displayed categorically to illustrate patterns, timepoint × autonomous selfing *p* < 0.005. Populations with autonomous selfing values over 0.50 are categorized as high autonomy (Appendix S1).

The average length of an individual's pollen‐collecting hairs at flower opening varied two‐fold among *C. americana* populations, ranging from 0.21 to 0.40 mm (Wald *χ* ^2^ = 152.25, p < 0.001, Appendix S2). A population's average pollen‐collecting hair length at flower opening increased with autonomous selfing ability (Figure 3A, F_1,13_ = 17.20, p = 0.001). Number of hairs on the circumference of the style cross‐section at flower opening also varied among populations ranging from 56 to 80 hairs on average (F_10,73_ = 2.16, p = 0.03, Appendix S2). However, most populations had similar numbers of pollen‐collecting hairs, with only 2 of the 11 populations different in post‐hoc Tukey tests. There was not a significant correlation between pollen‐collecting hair number and length (F_1,82_ = 1.95, p = 0.17) or pollen‐collecting hair number and autonomous selfing (F_1,9_ = 0.07, p = 0.80).

The number of pollen‐collecting hairs decreased sharply during the initial six hours after flower opening with approximately one‐third of all hairs fully retracting during this interval (Appendix S3). This was followed by a more gradual decrease in pollen‐collecting hair number across later timepoints (timepoint Wald *χ* ^2^ = 187.23, p < 0.001), with half of the hairs fully retracted by the final timepoint. The weighted average hair length that accounts for fully retracted hairs follows a similar pattern. Average hair length decreased 50% between zero and six hours and then decreased modestly across the remainder of a flower's first day (Wald *χ* ^2^ = 180.42, p < 0.001, Figure 3B). While populations with high levels of autonomous selfing started off with longer hairs, they retracted faster than those of populations with lower autonomous selfing potential and so were the same length 6 hours after floral anthesis (Figure 3C, timepoint × autonomous selfing interaction Wald *χ* ^2^ = 24.38, p < 0.001).

## DISCUSSION

Secondary pollen presentation is thought to have evolved to enhance outcrossing (Howell et al., 1993; Nyman, 1993a, b; Yeo, 1993; Westerkamp and Weber, 1997). We characterized intraspecific variation in pollen‐collecting hairs, a key trait in secondary pollen presentation in the Campanulaceae, and tested the trait's association with autonomous selfing ability in *Campanula americana*. We found longer pollen‐collecting hairs in populations with higher levels of autonomous selfing (Figure 3A). We also found that pollen‐collecting hairs in high‐autonomy populations had a faster retraction rate than those in low‐autonomy populations. As a result, six hours after floral anthesis, pollen‐collecting hairs were a similar length across all populations (Figure 3C), and therefore populations are not expected to differ in the degree to which pollen is available for outcrossing. These results suggest that initial pollen‐collecting hair length is driving differences in autonomous selfing ability across populations perhaps through increased pollen retention over time. In total, we demonstrate that pollen‐collecting hairs, a trait thought to have evolved to promote outcrossing, may also facilitate selfing, and therefore have a dual mating‐system function.

Pollen‐collecting hairs were longer at flower opening in *C. americana* populations with greater autonomous selfing potential than those with lower selfing potential. Previous work has shown that *C. americana* populations with elevated levels of autonomous selfing have more pollen on the stigmatic lobes at the beginning of female phase in the greenhouse (Koski et al., 2018), and greater pollen remaining following exposure to pollinators (Leibman et al., 2018) than populations with lower autonomous selfing potential. Longer pollen‐collecting hairs could underlie these results, possibly retaining pollen better by offering greater surface area and a more complex matrix in which to hold pollen. This is supported by the observation that pollen‐collecting hairs make it harder for insect visitors to collect pollen (Shetler, 1979; Nyman, 1993a, b; Makowski, personal observation). Additionally, longer hairs could hold onto more pollen when pollen is deposited from the anthers in bud, thereby facilitating increased pollen retention over time. In contrast, low‐autonomy populations have shorter pollen‐collecting hairs that may accumulate less pollen in bud or not hold on to it as well, decreasing their ability to retain pollen into the female phase.

Flowers in the high‐autonomy populations started off with longer pollen‐collecting hairs, but they retracted quickly such that they were the same length as low‐autonomy population flowers within six hours of anthesis (Figure 3C). The more rapid retraction of hairs in high‐autonomy populations may be associated with their shortened male phase (Koski et al., 2018). We anticipated that if hairs in the high‐autonomy populations were the same length as those in the low‐autonomy populations, they would retract more slowly than low‐autonomy populations, allowing pollen to be retained over a longer time window. Alternatively, if high‐autonomy populations had longer hairs than low‐autonomy populations, as we found, we predicted a comparable retraction rate would also allow for longer pollen retention. However, neither of these hypotheses were supported. Our finding of similar hair length six hours after anthesis suggests comparable opportunity for insect visitors to remove pollen and facilitate outcrossing regardless of autonomous selfing ability. Through developmental changes in length, pollen‐collecting hairs are a plastic phenotype that is associated with autonomous selfing ability while contributing to outcrossing in *C. americana*. Outcrossing rates in natural populations of *C. americana* are not associated with the population's autonomous selfing ability (Koski et al., 2019b), suggesting no tradeoff between hair traits and outcrossing ability. The association of pollen‐collecting hairs with both selfing and outcrossing suggests they experience selective pressure to promote opposing functions (Armbruster, 1988; Fenster and Martén‐Rodríguez, 2007).

Mixed‐mating systems are under dual selective pressures to optimize the ability to self and to outcross. When different selective pressures act on one trait, there can be multiple evolutionary outcomes including trade‐offs, common adaptive peaks, trait specialization, and non‐additive selection (Sahli and Connor, 2011). The evolution of selfing is characterized by a loss of floral characteristics that are associated with pollinator attraction, such a corolla size, color, and scent (Sicard and Lenhard, 2011), suggesting a trade‐off between outcrossing and selfing function. However, trade‐offs may be less likely for traits that promote selfing in mixed‐mating systems as they occur in flowers that are also adapted for outcrossing. For example, *C. americana* populations that differ in autonomous selfing potential have flowers with similar petal size (Makowski et al., 2024b), suggesting the evolution of selfing potential with no change in pollinator attraction. Alternatively, trait specialization may be the outcome of opposing selective pressures if evolution of selfing ability occurs without an impact to outcrossing ability. We found an increase in pollen‐collecting hair length that was associated with selfing alongside a compensation in retraction rate that likely facilitates outcrossing by making pollen more accessible to pollinators. Together these suggest specialization of a trait that contributes to mixed mating. This supports the idea that individual floral traits (Lankinen and Madjidian, 2011; Medrano et al., 2012) and changes across the floral life span (Fornoni et al., 2016) can influence multiple aspects of plant mating system without a trade‐off.

Mixed mating is thought to be favored when pollination is infrequent or unpredictable (Kalisz and Vogler, 2003; Ruan et al., 2009); therefore, it is critical to consider pollination adequacy to understand the evolution of mixed‐mating systems (Kalisz et al., 2011). Outcrossing rates in *C. americana* are not associated with autonomous selfing potential (Koski et al., 2019b). In addition, the contemporary pollinator environment does not explain clinal variation in autonomous selfing ability (Koski et al., 2017) and thus pollen‐collecting hair length. Rather, autonomous selfing ability is correlated with a population's distance from glacial refugia (Koski et al., 2019a), such that populations with longer post‐glacial colonization distances have greater autonomous selfing ability. Experimental colonization has demonstrated a benefit of autonomous selfing in mate‐limited environments (i.e., Baker's law, Makowski et al., 2024a), suggesting that historic demographic processes selected for selfing ability in *C. americana* (see also Griffin and Willi, 2014). As Baker (1967) stated, selfing provides “the chance of multiplying, so that [an individual] may form a population and even spread,” and the maintenance of the outcrossing function allows for “beneficial intercourse to follow,” suggesting an adaptive significance of mixed mating during colonization. Pollen‐collecting hairs in *C. americana* are an example of a trait with a dual mating‐system function that supports this ‘best of both worlds’ hypothesis of mixed‐mating systems (Stebbins, 1974; Lloyd, 1992).

## AUTHOR CONTRIBUTIONS

H.M., A.M.K., and L.F.G. conceived of the study. H.M., A.M.K., and E.S. collected data. K.L. led development of the machine learning model with assistance from H.M. H.M. led data analysis with assistance from K.L. and L.F.G. H.M. wrote the initial draft of the manuscript, with feedback from L.F.G. and K.L.

## Supporting information


**Appendix S1.** Geographic and autonomous selfing data for the 15 *Campanula americana* populations used in this study. Population, latitude, longitude, autonomous selfing ability, and year autonomous selfing ability was estimated are given.

**Appendix S2.** The average length (A) and number (B) of pollen‐collecting hairs in a cross section of styles of *C. americana* populations at floral opening.

**Appendix S3.** The number of *C. americana* pollen‐collecting hairs per style over the first day of floral anthesis.

## Data Availability

Data are available on Zenodo: https://doi.org/10.5281/zenodo.15803003.
