# Supplementary material for: Trait specialization facilitates autonomous selfing ability in a mixed‐mating plant
Source: Am J Bot. 2025 Sep 4;112(9):e70095. doi: 10.1002/ajb2.70095 (PMC12464461; doi:10.1002/ajb2.70095)
Supplement: Supplementary file 1 — Appendix S1. Geographic and autonomous selfing data for the 15 Campanula americana populations used in this study. Population, latitude, longitude, autonomous selfing ability, and year autonomous selfing ability was estimated are given. [file AJB2-112-e70095-s001.docx]

Makowski et al. – American Journal of Botany 2025 – Appendix S1

Appendix S1. Geographic and autonomy data for the 15 *Campanula americana* populations used in this study. Autonomy was quantified across multiple years (Koski et al., 2017; Koski et al., 2018; Makowski et al., 2024; H. Makowski, unpublished data). An asterisk in the experiment year column indicates the population was only used at timepoint 0 for the 2022 dataset. The red outline on the map indicates the species range from iNaturalist data.


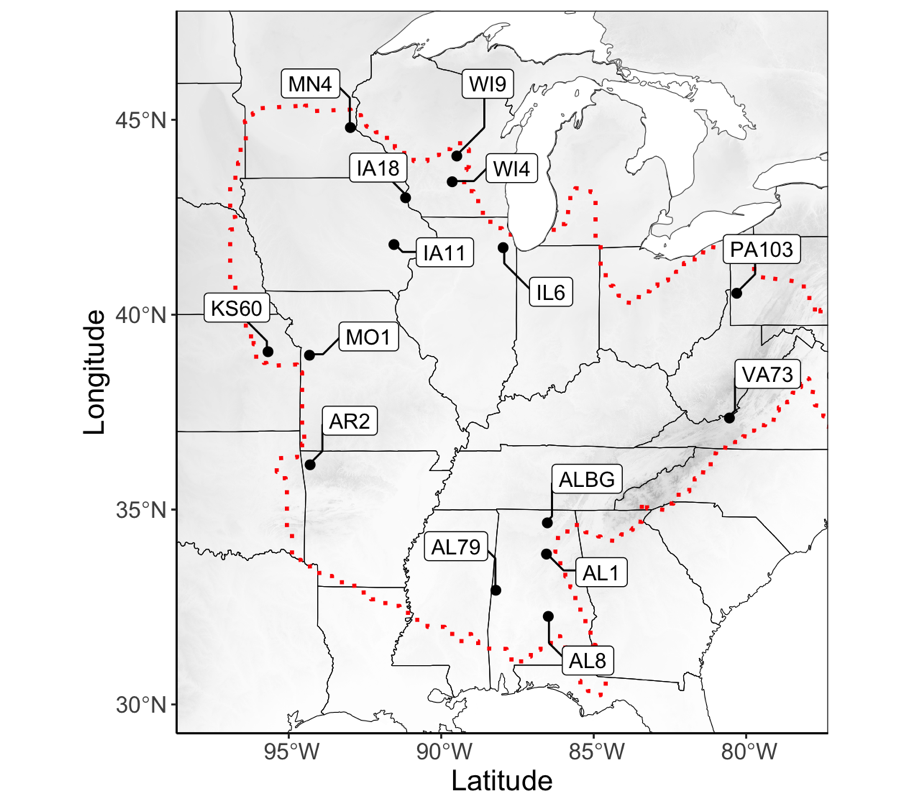


| **Population** | **Latitude** | **Longitude** | **Autonomy** | **Experiment year** |
| --- | --- | --- | --- | --- |
| AL8 | 32.26 | -86.49 | 0.45 | 2022* |
| AL79 | 32.93 | -88.21 | 0.40 | both |
| AL1 | 33.86 | -86.55 | 0.28 | 2022* |
| ALBG | 34.66 | -86.52 | 0.34 | 2022 |
| AR2 | 36.15 | -94.30 | 0.52 | 2022 |
| VA73 | 37.35 | -80.55 | 0.34 | both |
| MO1 | 38.96 | -94.32 | 0.36 | 2021 |
| KS60 | 39.05 | -95.68 | 0.60 | 2022 |
| PA103 | 40.55 | -80.31 | 0.28 | both |
| IL6 | 41.72 | -87.97 | 0.57 | both |
| IA11 | 41.80 | -91.55 | 0.60 | 2021 |
| IA18 | 43.00 | -91.17 | 0.55 | 2022 |
| WI4 | 43.41 | -89.64 | 0.68 | both |
| WI9 | 44.07 | -89.49 | 0.40 | 2021 |
| MN4 | 44.80 | -92.98 | 0.57 | both |
