# Supplementary material for: Trait specialization facilitates autonomous selfing ability in a mixed‐mating plant
Source: Am J Bot. 2025 Sep 4;112(9):e70095. doi: 10.1002/ajb2.70095 (PMC12464461; doi:10.1002/ajb2.70095)
Supplement: Supplementary file 2 — Appendix S2. The average length (A) and number (B) of pollen‐collecting hairs in a cross section of styles of C. americana populations at floral opening. [file AJB2-112-e70095-s002.docx]

Makowski et al. – American Journal of Botany 2025 – Appendix S2


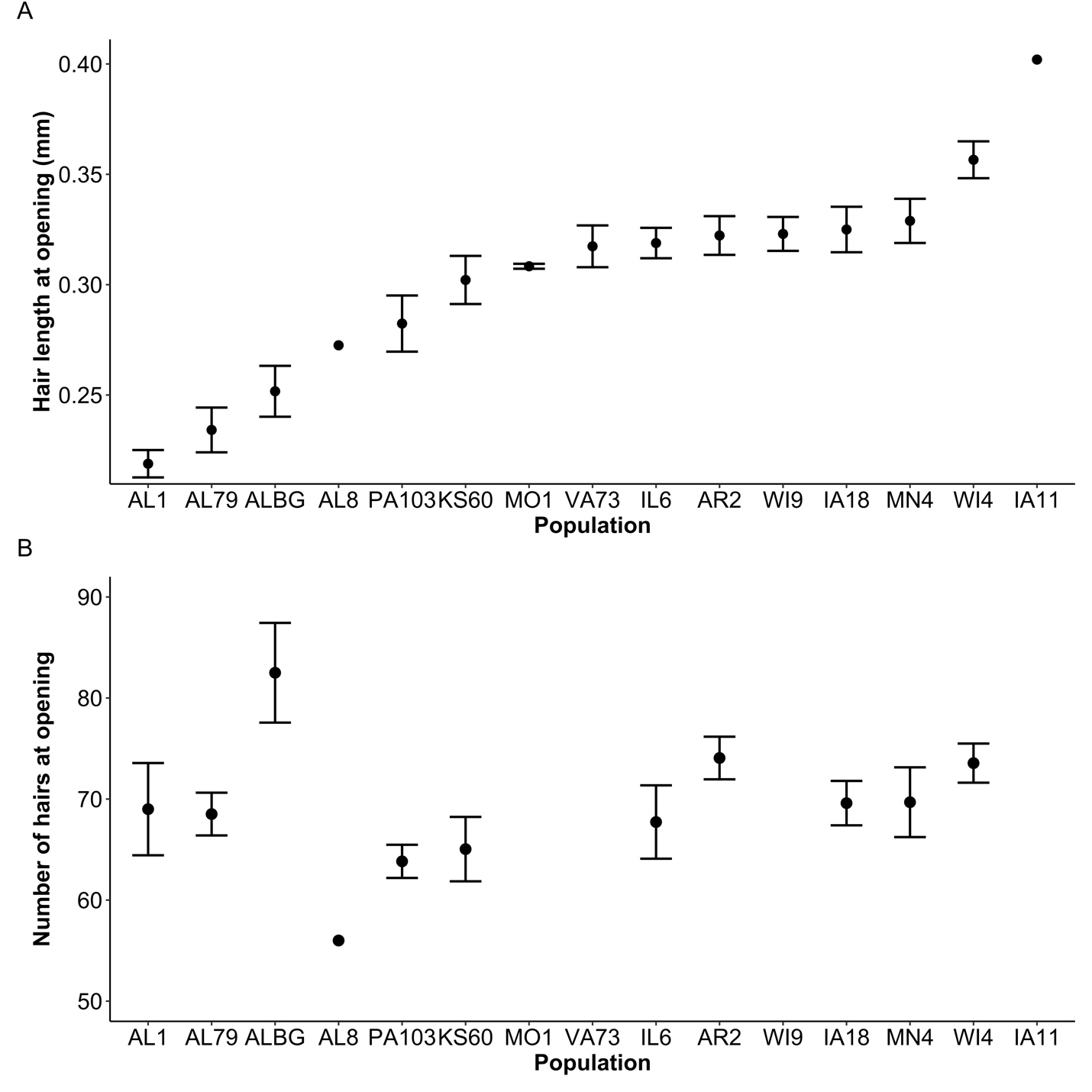


Appendix S2. The average length (A) and number (B) of pollen collecting hairs in a cross section of styles of *C. americana* populations at floral opening. Populations are ordered by ascending hair length; points represent population means and bars represent SE. Four populations do not have data for hair number.
