# Supplementary material for: Trait specialization facilitates autonomous selfing ability in a mixed‐mating plant
Source: Am J Bot. 2025 Sep 4;112(9):e70095. doi: 10.1002/ajb2.70095 (PMC12464461; doi:10.1002/ajb2.70095)
Supplement: Supplementary file 3 — Appendix S3. The number of C. americana pollen‐collecting hairs per style over the first day of floral anthesis. [file AJB2-112-e70095-s003.docx]

Makowski et al. – American Journal of Botany 2025 – Appendix S3


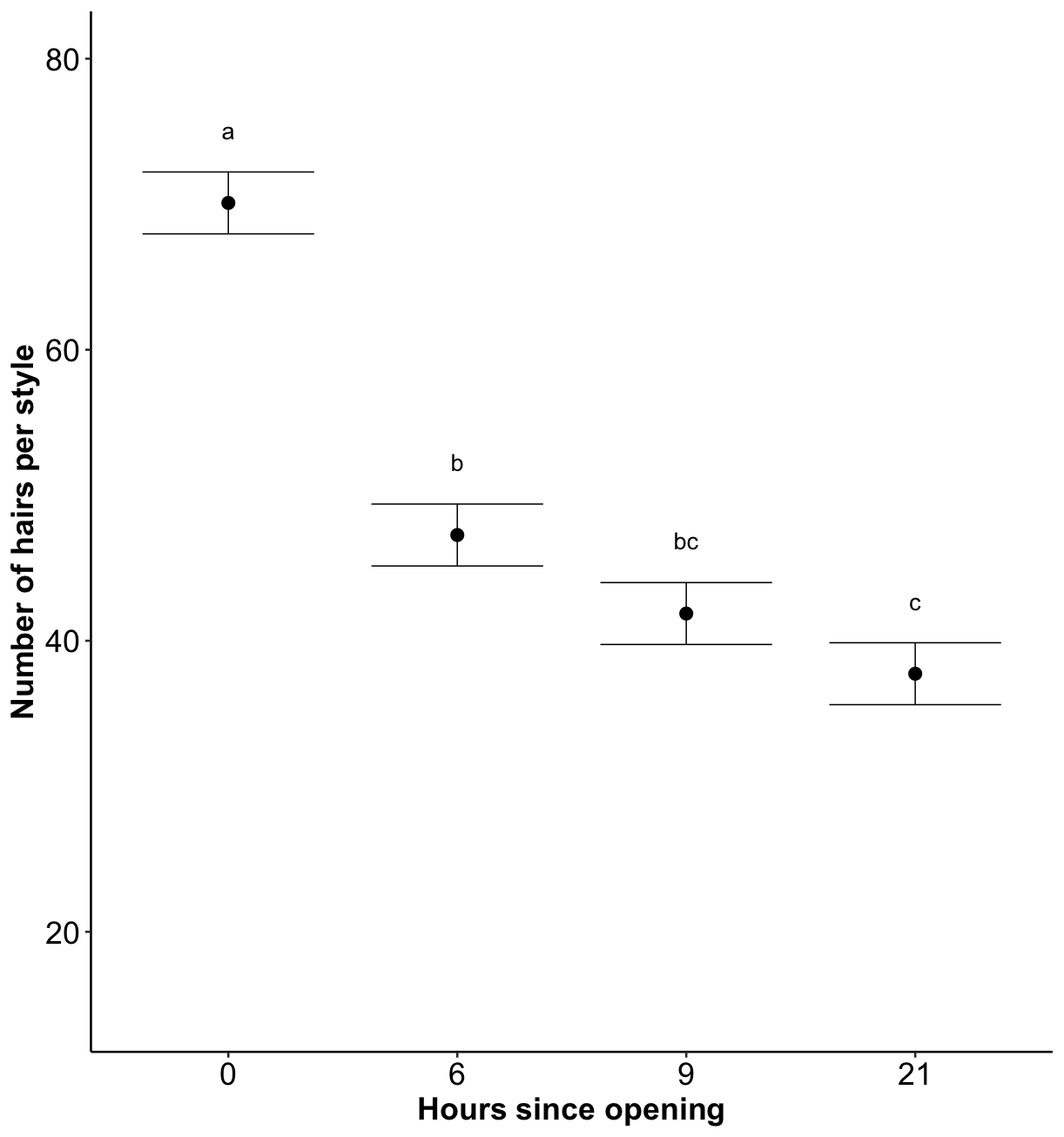


Appendix S3. The number of *C. americana* pollen-collecting hairs per style over the first day of floral anthesis. Points represent averages across nine populations at each timepoint; error bars are SE. Letters signify statistical difference from post-hoc Tukey test.
